# Elevational patterns of microbial species richness and evenness across climatic zones and taxonomic scales

**DOI:** 10.1002/ece3.10594

**Published:** 2023-10-09

**Authors:** Zhengyi Huang, Yangui Su, Sinuo Lin, Guopeng Wu, Hao Cheng, Gang Huang

**Affiliations:** ^1^ School of Geographical Sciences, School of Carbon Neutrality Future Technology Fujian Normal University Fuzhou China

**Keywords:** biodiversity, climatic zones, drivers, elevational patterns, microorganisms, taxonomic scales

## Abstract

Understanding the elevational patterns of soil microbial diversity is crucial for microbial biogeography, yet the elevational patterns of diversity across different climatic zones, trophic levels, and taxonomic levels remain unclear. In this study, we investigated the elevational patterns of species richness, species evenness and the relationship between species richness and evenness (RRE) in the forest soil bacterial and fungal communities and individual phyla across three climatic zones (tropical, subtropical, and cold temperate). Our results revealed that soil bacterial richness (alpha diversity) decreased with elevation, while fungal richness exhibited a hump‐shaped pattern in the tropical and cold‐temperate forests. Elevational patterns of evenness in bacterial and fungal communities showed the hump‐shaped pattern across climatic zones, except for bacterial evenness in the tropical forest. Both bacterial and fungal richness and evenness were positively correlated in the subtropical and cold‐temperate forests, while negatively correlated for bacteria in the tropical forest. The richness and evenness of soil microorganisms across different regions were controlled by climatic and edaphic factors. Soil pH was the most important factor associated with the variations in bacterial richness and evenness, while mean annual temperature explained the major variations in fungal richness. Our results addressed that the varieties of elevational patterns of microbial diversity in climatic zones and taxonomic levels, further indicating that richness and evenness may respond differently to environmental gradients.

## Graphical abstract

A continental‐scale investigation on the elevational patterns of forest soil microbial diversity was involved. The elevation patterns of microbial diversity varied in climate zones and taxonomic levels. The relationship between richness and evenness was inconsistent in climate zones and taxonomic levels. pH caused bacterial diversity variation, and mean annual temperature caused fungal richness variation.

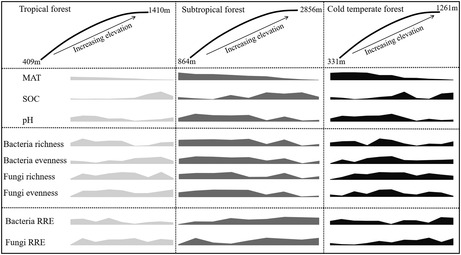

## 1 INTRODUCTION

Soil microorganisms play an important role in ecosystem functions and services such as ecosystem productivity, biogeochemical cycles, and climate regulation (Escalas et al., 2019; Khlifa et al., 2017; Saitta et al., 2018). Microorganisms are the most diverse and abundant organisms on the Earth, and they have important metabolic functions, including the decomposition of soil organic matter, regulating carbon stocks, and nutrient cycling (Sánchez‐Galindo et al., 2021). Changes in soil microbial diversity and community composition can affect ecosystem functions and the associated services (Koranda et al., 2013; Reese et al., 2018). Soil microbial diversity and community composition vary with respect to changes in geographical gradients (Wang et al., 2017; Xu et al., 2023). Microbes are known to exhibit specific adaptations to these varying conditions (Wani et al., 2022). By studying their distribution patterns, researchers can gain a better understanding of microbial biodiversity, their ability to adapt to different ecological niches, and their responses to climate change (Yang et al., 2023). Soil microbial biogeography is important for understanding biogeochemical cycling and climate feedback of terrestrial ecosystems (Saitta et al., 2018).

The elevational pattern of biological distribution is not only a fundamental aspect of physical adaptation but also has important implications for predicting ecosystem responses to climate change (Looby & Martin, 2020; Rahbek et al., 2019). Temperature, precipitation, and other factors at different elevations of the mountain ecosystem gradually change along elevation (Sundqvist et al., 2013; Wang et al., 2022). Therefore, the elevational patterns of biodiversity and adaptability could reflect the responses of ecosystem to future climatic scenarios (Frac et al., 2018). Yet, despite many practical studies have revealed elevational patterns of the diversity in vertebrates, macroinvertebrates, and plants (Currie & Paquin, 1987; McCain, 2005; Renaud et al., 2009), elevational patterns of soil microorganisms have limited empirical investigation, and they are inconsistent in previous studies, such as no obvious pattern (Fierer et al., 2011; Shen et al., 2013), a decline (Bahram et al., 2012; Bryant et al., 2008; Ji et al., 2022; Luo et al., 2019), hump‐shaped (Miyamoto et al., 2014; Ren et al., 2021; Zhang et al., 2014), and U‐shaped (Li et al., 2016). Those inconsistencies maybe related to the differences in trophic levels (fungi, bacteria, etc.), taxonomic groups (phylum level), and climatic zones (Wang et al., 2017).

Although previous studies have investigated the elevational patterns and major determinants of soil microbial communities, only a few surveys have been conducted simultaneously across different climatic zones (Wang et al., 2017; Xu et al., 2023). The range of environmental differences along elevation in high‐latitude climatic zones may have exceeded the physiological threshold of some microorganisms. Moreover, the adaptability and environmental resistance in bacterial and fungal communities, and different microbial taxonomic groups are different (Xie et al., 2023; Yeh et al., 2019). Until now, a few studies have explored the general elevational patterns of microorganisms, and the relative impacts of climatic and soil properties on microbial communities and taxonomic groups in different climatic zones have been largely overlooked (Binkenstein et al., 2018; Wang et al., 2017).

Most studies on diversity focused on species richness, far less is known about evenness (Fauth et al., 1989; Lopez‐Angulo et al., 2020; Wang et al., 2011). Species evenness is another key diversity component, which describes the distribution of relative abundance in a community (Magurran, 2021). Most studies have used species richness as a proxy to study patterns and drivers of biodiversity, which could be a drawback because evenness can be an important component of diversity, while changes in richness alone do not reflect diversity well, and different processes may shape both aspects of biodiversity (Wang et al., 2017; Wilsey et al., 2005; Wilsey & Potvin, 2000). For example, one study suggested that bacterial richness and evenness respond differently to environmental gradients in elevation (Wang et al., 2017). Evenness could be associated with ecosystem functions, such as primary productivity (Hillebrand et al., 2008). Therefore, it is important to study elevational patterns of evenness in order to fully understand the effects of climate change on biodiversity distribution (Wang et al., 2017). In addition, the relationship between species richness and evenness (RRE), which reflects the dependent of evenness on richness, as well as their divergent responses, remains a controversial issue in ecology (Wang et al., 2017).

Exploring how the elevational patterns of species richness, species evenness, and RRE responds to climatic zones, trophic levels and taxonomic levels can help us to more comprehensively understand soil microbial community diversity distribution and influencing factors, which is especially important for the potential functions provided by microbial communities, such as carbon and nitrogen retention (Liu et al., 2018). In this study, we examined the elevational patterns of the richness and evenness of bacteria and fungi, and RRE in three mountain forests from different climatic zones (Jianfengling tropical, Shennongjia subtropical, and Xing'anling cold temperate); furthermore, we tested the congruence of elevational patterns of richness, evenness, and RRE in climatic zones and taxonomic groups. Finally, we distinguished the relative importance of climatic and edaphic factors on the variations of species richness, evenness, and RRE in entire community and individual phyla. We hypothesized that (1) climatic zones affect the elevational patterns of bacterial and fungal richness and evenness in forest soils, as observed for the microorganisms in mountain streams (Wang et al., 2017); (2) the RRE varies in climatic zones, trophic levels, and taxonomic levels (Soininen et al., 2012); and (3) soil properties are important factors affecting the diversity of bacterial and fungal communities along the elevation gradients.

## 2 MATERIALS AND METHODS

### 2.1 Site description and soil sampling

**FIGURE 1:**
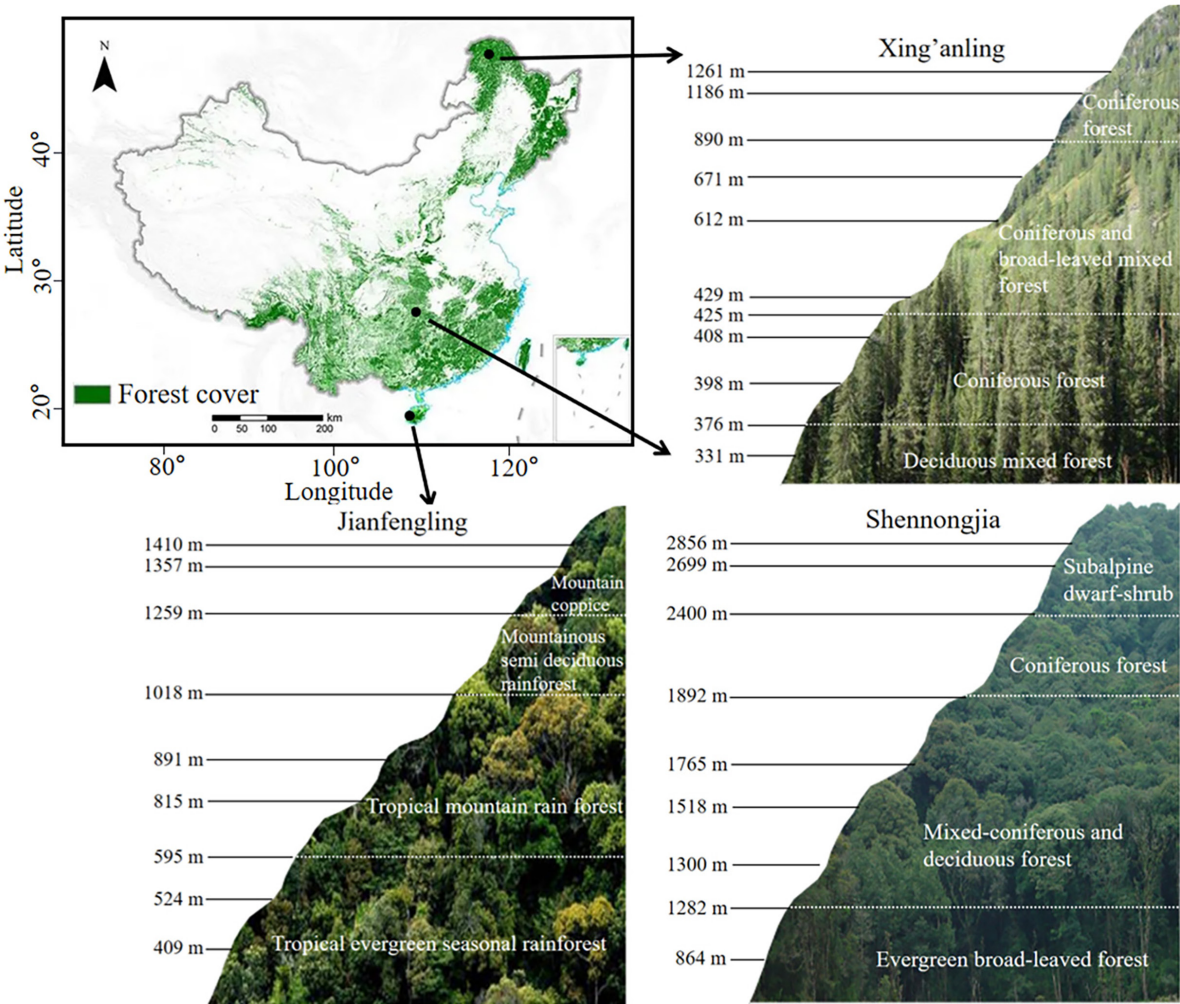
Overview map of the study area and distribution map of sampling points.

In 2021, soil samples were obtained from three distinct climatic zones [Jianfengling (JFL), Shennongjia (SNJ), and Xing'anling (XAL)] in China along the mountainsides. We set nine elevations in the JFL, nine elevations in the SNJ, and 11 elevations in the XAL, based on the climate and soil properties. JFL has a tropical island monsoon climate, with a mean annual temperature of 24.5°C and annual precipitation of 1600–2600 mm. SNJ has a north subtropical monsoon climate, with a mean annual temperature of 12°C and annual precipitation of 800–2500 mm. The XAL has a cold‐temperate climate properties, with a mean annual temperature of −1.2°C and annual precipitation of 360–500 mm. Soil was sampled from each site along the elevation gradient in each mountain. At each elevation, a representative soil sample was collected composed of five subsamples from five sampling points (10 m × 10 m). The detailed information of the elevational sites is listed in Table S1 and Figure 1. After removing litter and surface debris, we collect soil samples from the four corners and centers of each plot by using a shovel to excavate soil to a depth of 0–20 cm. The soil samples in each plot were then placed in sterile plastic bags and transported to the laboratory. In the laboratory, all samples were passed through a 2 mm soil sieve, and the soils of each plot were combined, thoroughly mixed, and divided into three parts: Portions of fresh soil were stored at −80°C for DNA extraction and at 4°C for microbial biomass analyses, while the remainder was air‐dried and stored at room temperature prior to soil physical and chemical analysis.

### 2.2 Measurements of soil physicochemical properties

Soil pH was measured by a pH meter (Starter 300) after shaking a soil–water (1:2.5 w/v) suspension for 30 min (Chen et al., 2019). Soil organic carbon (SOC) and total nitrogen (TN) were measured with a combustion analyzer (Elemental Analyzer Vario ELIII). Soil total phosphorus (TP) was determined with a flow injection auto‐analyzer (Skalar San++) following digestion with H_2_SO_4_–HClO_4_ (Huang et al., 2011). Soil microbial biomass carbon (MBC), nitrogen (MBN), and phosphorus (MBP) were measured by fumigation–extraction method (Brookes et al., 1985). MBC and MBN were extracted using 0.5 M K_2_SO_4_, and MBP was extracted using 0.5 M NaHCO_3_ (pH = 8.5). MBC was measured on a TOC analyzer (TOC VPH/CPN, Shimadzu), and MBN and MBP were measured using a flow injection auto‐analyzer (Skalar San++). MBC, MBN, and MBP were corrected with an extraction efficiency factor of 0.45, 0.54, and 0.40, respectively (Jeannotte et al., 2004; Joergensen et al., 2011). The mean annual temperature (MAT) and mean annual precipitation (MAP) data were obtained from previous literatures (Guo et al., 2020; Wu et al., 2023; Yang et al., 2014).

### 2.3 DNA extraction and Illumina MiSeq sequencing of bacterial and fungal communities

The total DNA of the soil was extracted using the PowerSoil DNA Isolation Kit from MOBIO company. The quality of the DNA was assessed using 1% agarose gel electrophoresis. The bacterial 16S and fungal ITS genes were amplified using specific primers. For the bacterial amplification, the V3–V4 region was targeted using primers 338F (5′‐ACTCCTACGGGAGGCAGCAG‐3′) and 806R (5′‐GGACTACHVGGGTWTCTAAT‐3′; Hong et al., 2015). For the fungal amplification, the ITS2 region was targeted using primers ITS3F (5′‐GCATCGATGAAGAACGCAGC‐3′) and ITS4R (5′‐TCCTCCGCTTATTGATATGC‐3′; Orgiazzi et al., 2012), with a unique 12 bp barcode added at the 5′ end of the reverse primer to enable sample identification. Each sample had its own unique barcode, and the PCR mixture (25 μL) contained 1 × PCR buffer, 1.5 mM MgCl_2_, 0.4 mM deoxynucleotide triphosphate, each primer of 1.0 μM, 0.5 U ExTaq (Takara), and 10 ng of soil genomic DNA. The PCR amplification procedure consisted of an initial denaturation step at 98°C for 1 min, followed by 30 cycles at 98°C for 10 s, 50°C for 30 s, 72°C for 30 s, and a final extension at 72°C for 5 min. The purified PCR products were obtained using the GeneJET™ Gel Extraction Kit (Thermo Scientific), and the TruSeq DNA kit was used to mix all purified PCR products in equimolar amounts for library construction. The Illumina MiSeq platform (Illumina Inc.) from Beijing Baimaike Biotechnology Co., Ltd. was used for sequencing. Raw reads were deposited into the National Genomics Data Center Nucleotide Sequence Database (Accession Number: PRJCA015582).

Sequence processing, clustering, taxonomic assignments, and biodiversity calculations were performed with the QIIME (V1.7.0, http://qiime.org/index.html). The samples were sequenced in equimolar amounts, and paired‐end sequencing (2 × 300 bp) was performed according to the standard protocols of Baimaike Biotechnology Co., Ltd. In the initial step, the sequences were de‐multiplexed, and the primer and barcode sequences were removed. Subsequently, sequences with high quality, defined as those with a length > 260 bp, lacking ambiguous ‘N’ bases, and an average base quality score > 30, were retained for downstream analyses. Operational taxonomic units (OTUs) were generated using an open‐reference OTU picking protocol, where the sequences were clustered against the Greengenes database, with a 97% similarity cutoff. Taxonomic assignments were made by performing BLAST searches against the SILVA bacterial and UNITE fungal ITS databases for the 16S and ITS gene sequences, respectively.

### 2.4 Statistical analysis

Taxonomic diversity (OTU richness, Pielou's evenness) was estimated in QIIME with ‘alphadiversity.py’ script (Shen et al., 2019). Although it is mathematically impossible to decompose diversity into truly independent richness and evenness components, Pielou's evenness, is calculated as *J* = *H*/log(*S*) (where *H* is the Shannon–Weaver diversity index and *S* is the number of species), is a good measure of distribution of relative abundance in a community (Jost, 2010). Therefore, we selected species richness and Pielou's evenness as biodiversity indicators reflecting two aspects of community biodiversity. Linear mixed‐effects modeling was used to evaluate the differences in soil microbial diversity (richness and evenness) between elevations in different climatic regions. The fixed factors were the elevations, and the random factor was the climatic zone. We finally selected 14 microbial phyla, including the top 10 bacterial phyla and the top 4 fungal phyla in relative abundance, which were present in more than 60% of the samples and in samples from all three climatic zones (Yeh et al., 2019). The relationship between richness, evenness, and elevation, and the relationship between richness and evenness (RRE) at community level and phylum level were investigated by linear and quadratic models. We selected the better model based on the lower value of Akaike's information criterion (Yamaoka et al., 1978).

To determine the relative importance of different environmental factors in explaining the richness and evenness of bacterial and fungal communities, we performed a random forest analysis (Trivedi et al., 2016). We considered the following explanatory variables: mountain (as a categorical variable), soil pH, soil organic carbon (SOC), total nitrogen (TN), total phosphorus (TP), mean annual temperature (MAT), mean annual precipitation (MAP), soil organic carbon to total nitrogen ratio (C/N), soil organic carbon to total phosphorus ratio (C/P), and total nitrogen to total phosphorus ratio (N/P).

We assessed statistical dependence between the explanatory variables using Spearman rank correlation coefficients (rs). SOC was highly correlated with TN (rs = 0.70); thus, TN was excluded from further analyses. C/P was highly correlated with N/P (rs = 0.70); thus, N/P was excluded from further analyses. To estimate the importance of these environmental indices, we used percentage increases in the mean squared error (MSE) of variables, where higher MSE% values imply more important variables (Breiman, 2001). We assessed the significance of the models and cross‐validated *R* ^2^ values using 5000 permutations of the response variable, using the ‘A3’ package. Similarly, we assessed the significance of each predictor on the response variables using the ‘rfPermute’ package. We used Pearson's correlations to determine how environmental variables influence the biodiversity of the entire bacterial and fungal communities and at the phylum level using the R ‘cor.test’ function. For the relationship between community dissimilarity and elevation, non‐metric multidimensional scaling analysis (NMDS) was performed. Finally, we correlated Pielou's evenness with the richness between samples to obtain the correlation coefficient (RRE) between evenness and richness. As an effect size in the analyses, we used the Fisher transformed correlation coefficient *r*_*z*_ because it normalizes the distribution of the correlation coefficient. It was calculated as *r*_*z*_ = 0.5 × ln[(1 + *r*)/(1 − *r*)], where *r* is the coefficient of correlation between evenness and richness. All of the above analyses were performed using R version 4.1.3.

## 3 RESULTS

### 3.1 The elevational patterns of soil microbial richness at different taxonomy levels

**FIGURE 2:**
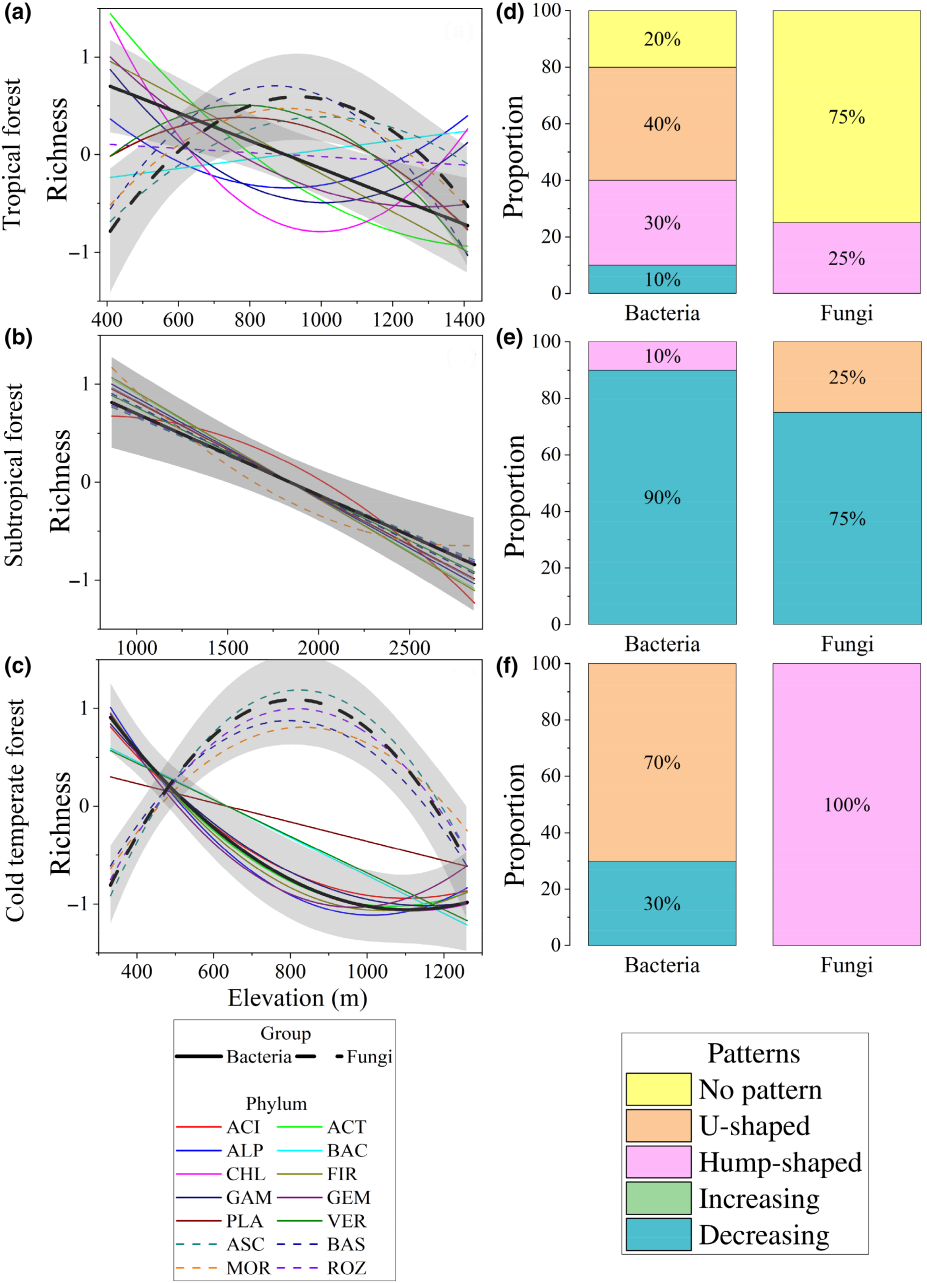
Elevational patterns in richness for microbial phyla. Three climate zones, that is, tropical forest (a), subtropical forest (b), and cold‐temperate forest (c), were considered for each bacterial or fungal phylum and their whole community. The richness trends of bacterial and fungal whole communities are presented in black solid and dotted lines, respectively, and the trends of phylum richness are shown with colored lines. Species richness are scaled as mean = 0 and SD = 1 for better visualization. More detailed elevational patterns in biodiversity are shown in Figure S1. The relative proportions of different patterns at phylum level were presented in the right panels of tropical forest (d), subtropical forest (e), and cold‐temperate forest (f). Different colors represent different categories of patterns. The trends along elevations were modeled with both linear and quadratic models. The better model was selected based on the lower value of Akaike's information criterion. ACI, Acidobacteria; ACT, Actinobacteria; ALP, Alphaproteobacteria; ASC, Ascomycota; BAC, Bacteroidetes; BAS, Basidiomycota; CHL, Chloroflexi; FIR, Firmicutes; GAM, Gammaproteobacteria; GEM, Gemmatimonadetes; MOR, Mortierellomycota; PLA, Planctomycetes; ROZ, Rozellomycota; VER, Verrucomicrobia.

Elevation has a significant effect on microbial diversity (Table 1). The richness of entire soil bacterial community in tropical and subtropical forests decreased linearly with elevation (Figure 2a,b, *p* < .05), and exhibited an asymptotic decreasing pattern in the cold‐temperate forest (Figure 2c). At the phyla level, 40% and 70% of bacterial phyla showed a U‐shaped pattern with elevation in the tropical and cold‐temperate forests, respectively, whereas 90% of bacterial phyla showed the linear declining pattern in the subtropical forest (Figure 2d–f). Some bacterial phyla in the tropical forest also showed hump‐shaped or U‐shaped patterns, while others in the cold‐temperate forest showed asymptotic decreasing pattern.

**TABLE 1:** Linear mixed‐effects modeling was used to evaluate the effect of elevation on microbial diversity (with climate zone as a random effect).

	Estimate	Std. error	*t*‐Value	*p*‐Value
(a) *Bacterial richness*				
Intercept	0.0011	0.011	9.788	0
Elevation	−0.1622	0.071	−2.294	<.001
Elevation^2^	−0.00002	0.00002	−0.982	<.001
*Random effects variance*				
Climatic zone	32,755			
Residual	17,689			
Marginal *R* ^2^	.380			
Conditional *R* ^2^	.720			
(b) *Bacterial evenness*				
Intercept	0.634	0.038	16.537	0
Elevation	0.0002	0.00004	4.456	<.001
Elevation^2^	−0.0000001	0.0000001	−6.979	<.001
*Random effects variance*				
Climatic zone	0.00294			
Residual	0.00744			
Marginal *R* ^2^	.390			
Conditional *R* ^2^	.514			
(c) *Fungal richness*				
Intercept	0.056	0.026	2.207	0
Elevation	0.090	0.039	2.294	<.001
Elevation^2^	−0.0001	0.00001	−4.175	<.001
*Random effects variance*				
Climatic zone	0.00294			
Residual	0.00744			
Marginal *R* ^2^	.016			
Conditional *R* ^2^	.961			
(d) *Fungal evenness*				
Intercept	0.5103	0.0572	8.929	0
Elevation	0.0002	0.00004	5.252	<.001
Elevation^2^	−0.0000001	0.0000001	−8.026	<.001
*Random effects variance*				
Climatic zone	0.01074			
Residual	0.01024			
Marginal *R* ^2^	.378			
Conditional *R* ^2^	.622			

*Note*: Marginal *R* ^2^ is explained by all the fixed effects together; conditional *R* ^2^ is explained by both fixed and random effects. We checked for spatial auto‐correlation of the residuals for each model using Moran's *I* index (not significant in all cases).

For fungal richness, the entire community showed a hump‐shaped pattern with increasing elevation in the tropical and cold‐temperate forests, and linearly decreased in the subtropical forest (Figure 2a–c). Most of the fungal phyla in the tropical forest (75%) displayed no obvious pattern, whereas 75% of the phyla in the subtropical forest showed linear declining pattern. All of the fungal phyla in the cold‐temperate forest showed a hump‐shaped pattern (Figure 2d–f).

### 3.2 The elevational patterns of soil microbial evenness at different taxonomy levels

**FIGURE 3:**
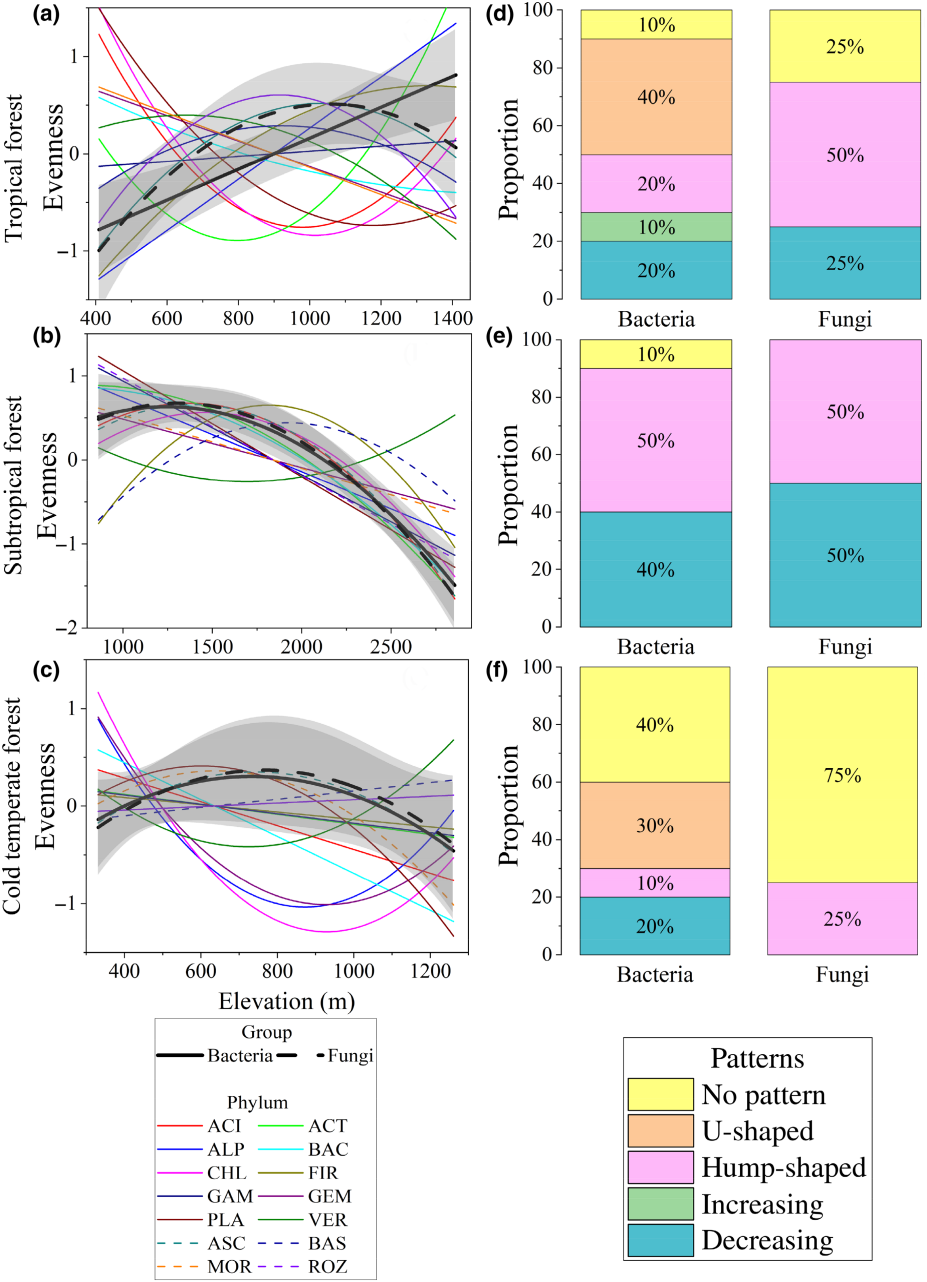
Elevational patterns in evenness for microbial phyla. Three climate zones, that is, tropical forest (a), subtropical forest (b), and cold‐temperate forest (c), were considered for each bacterial or fungal phylum and their whole community. The evenness trends of bacterial and fungal whole communities are presented in black solid and dotted lines, respectively, and the trends of phylum evenness are shown with colored lines. Species evenness are scaled as mean = 0 and SD = 1 for better visualization. More detailed elevational patterns in biodiversity are shown in Figure S1. The relative proportions of different patterns at phylum level were presented in the right panels of tropical forest (d), subtropical forest (e), and cold‐temperate forest (f). Different colors represent different categories of patterns. The trends along elevations were modeled with both linear and quadratic models. The better model was selected based on the lower value of Akaike's information criterion. ACI, Acidobacteria; ACT, Actinobacteria; ALP, Alphaproteobacteria; ASC, Ascomycota; BAC, Bacteroidetes; BAS, Basidiomycota; CHL, Chloroflexi; FIR, Firmicutes; GAM, Gammaproteobacteria; GEM, Gemmatimonadetes; MOR, Mortierellomycota; PLA, Planctomycetes; ROZ, Rozellomycota; VER, Verrucomicrobia.

The evenness of the entire soil bacterial community increased linearly with elevation in the tropical forest (Figure 3a, *p* < .05) and exhibited a hump‐shaped pattern in both the subtropical and cold‐temperate forests (Figure 3b,c). At the phylum level, 20%, 50%, and 10% of bacterial phyla showed a hump‐shaped pattern with elevation in the tropical, subtropical, and cold‐temperate forests, respectively (Figure 3d–f).

Regarding fungal evenness, the entire community displayed a hump‐shaped pattern with an increase in elevation in all three forests: tropical, subtropical, and cold temperate (Figure 3a–c). Among the fungal phyla, 50% in the tropical forest showed hump‐shaped pattern, 50% in the subtropical forest displayed linear decreasing pattern, and 75% in the cold‐temperate forest exhibited no obvious pattern (Figure 3d–f).

### 3.3 The relationship between soil microbial richness and evenness at different taxonomy levels

**FIGURE 4:**
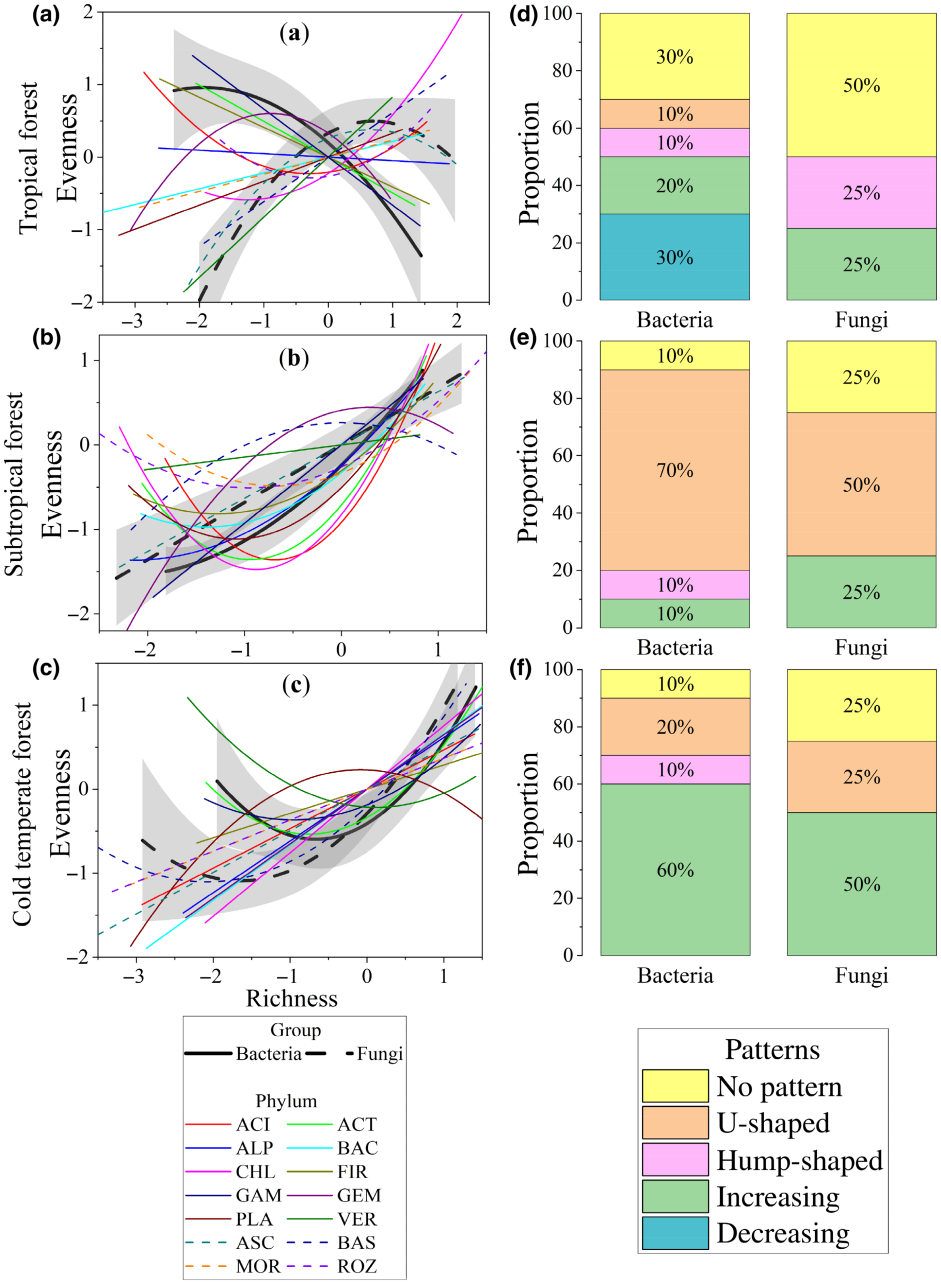
Elevational patterns in relationship between richness and evenness (RRE) for microbial phyla. Three climate zones, that is, tropical forest (a), subtropical forest (b), and cold‐temperate forest (c), were considered for each bacterial or fungal phylum and their whole community. The RRE trends of bacterial and fungal whole communities are presented in black solid and dotted lines, respectively, and the trends of phylum RRE are shown with colored lines. Species RRE are scaled as mean = 0 and SD = 1 for better visualization. More detailed elevational patterns in biodiversity are shown in Figure S1. The relative proportions of different patterns at phylum level were presented in the right panels of tropical forest (d), subtropical forest (e), and cold‐temperate forest (f). Different colors represent different categories of patterns. The trends along elevations were modeled with both linear and quadratic models. The better model was selected based on the lower value of Akaike's information criterion. ACI, Acidobacteria; ACT, Actinobacteria; ALP, Alphaproteobacteria; ASC, Ascomycota; BAC, Bacteroidetes; BAS, Basidiomycota; CHL, Chloroflexi; FIR, Firmicutes; GAM, Gammaproteobacteria; GEM, Gemmatimonadetes; MOR, Mortierellomycota; PLA, Planctomycetes; ROZ, Rozellomycota; VER, Verrucomicrobia.

The RRE in the entire soil bacterial community showed a negative association in the tropical forest, whereas a positive association was observed in the subtropical and cold‐temperate forests (Figure 4a–c, *p* < .05). At the phyla level, 20% and 60% of bacterial phyla exhibited a linear increasing pattern in the tropical and cold‐temperate forests, respectively, while 70% of bacterial phyla showed a U‐shaped pattern in the subtropical forest (Figure 4d–f). RRE in Acidobacteria displayed no significant relationship in the tropical forest, a U‐shaped pattern in the subtropical forest, and a positive linear pattern in the cold‐temperate forest. Similarly, Actinobacteria showed a negative linear relationship between richness and evenness in the tropical forest, and a U‐shaped pattern in the subtropical and cold‐temperate forests (Figure S1).

Regarding fungi, the relationship between richness and evenness showed a significant positive quadratic pattern in the tropical and cold‐temperate forests, and a positive linear pattern in the subtropical forest (Figure 4a–c). Ascomycota exhibited a hump‐shaped relationship between richness and evenness in the tropical forest, whereas a positive linear pattern was observed in the subtropical and cold‐temperate forests. Basidiomycota displayed a positive relationship between richness and evenness in the tropical and cold‐temperate forests, and no significant pattern was observed in the subtropical forest (Figure S1).

### 3.4 Variations of the relationship between soil microbial richness and evenness (RRE) in climatic zones (Z), trophic levels (T), and taxonomic levels (P)

**FIGURE 5:**
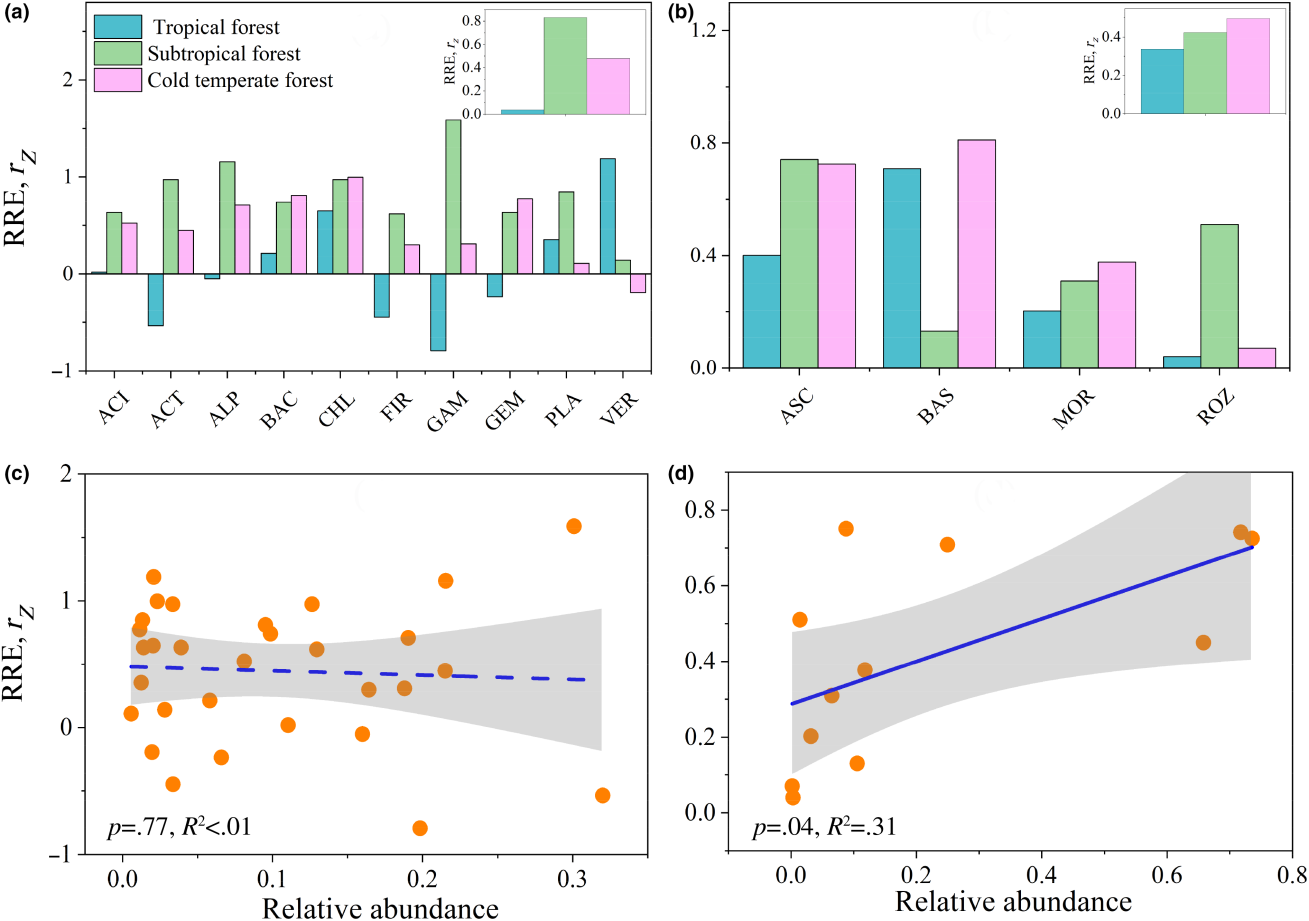
The RRE (*r*_*z*_) values in the ten phyla of bacteria (a) and four phyla of fungi (b) in three climate regions. The upper right corner of the image (a, b) shows the RREs of each climate zone. The observed values of the relationship between species richness and evenness (RRE, *r*_*z*_) along (c) relative abundance of bacteria and (d) relative abundance of fungi. ACI, Acidobacteria; ACT, Actinobacteria; ALP, Alphaproteobacteria; ASC, Ascomycota; BAC, Bacteroidetes; BAS, Basidiomycota; CHL, Chloroflexi; FIR, Firmicutes; GAM, Gammaproteobacteria; GEM, Gemmatimonadetes; MOR, Mortierellomycota; PLA, Planctomycetes; ROZ, Rozellomycota; VER, Verrucomicrobia.

The average RRE of bacteria (*r*_*z*_ = 0.45) in the three climatic zones was higher than that of fungi (*r*_*z*_ = 0.42; *p* < .05). At the individual forest level, the average RRE (*r*_*z*_) in the subtropical forest was found to be 0.83, which was higher than that in the tropical (*r*_*z*_ = 0.04) and cold‐temperate forests (*r*_*z*_ = 0.48) for bacteria (Figure 5a,b; *p* < .05). However, for fungi, the average RRE (*r*_*z*_) in the cold‐temperate forest was higher (*r*_*z*_ = 0.50) compared to that in the tropical (*r*_*z*_ = 0.34) and subtropical forests (*r*_*z*_ = 0.42; Figure 5a,b; *p* < .05). In terms of relative abundance, bacterial relative abundance did not show any significant variation with RRE (*p* = .77, *R* ^2^ < .01), while fungal relative abundance exhibited a positive relationship with RRE (*p* = .04, *R* ^2^ = .31; Figure 5c,d).

### 3.5 Driving forces of the soil microbial richness and evenness along the elevational gradients

**FIGURE 6:**
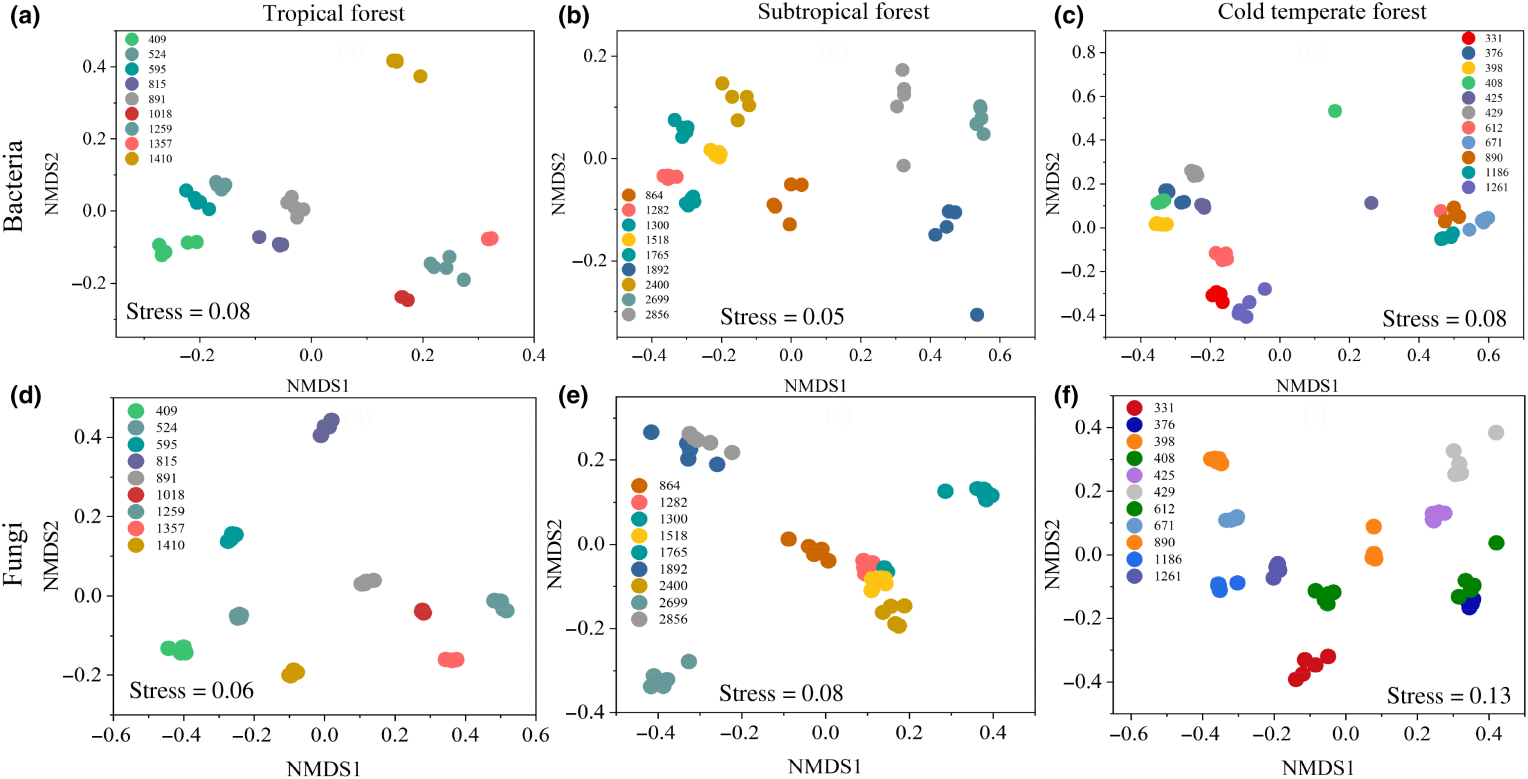
Non‐metric multidimensional scaling (NMDS) ordinations of the dissimilarities of the bacterial (a–c) and fungal (d–f) communities at different climatic regions. Sites were coded with different colors according to the elevations.

**FIGURE 7:**
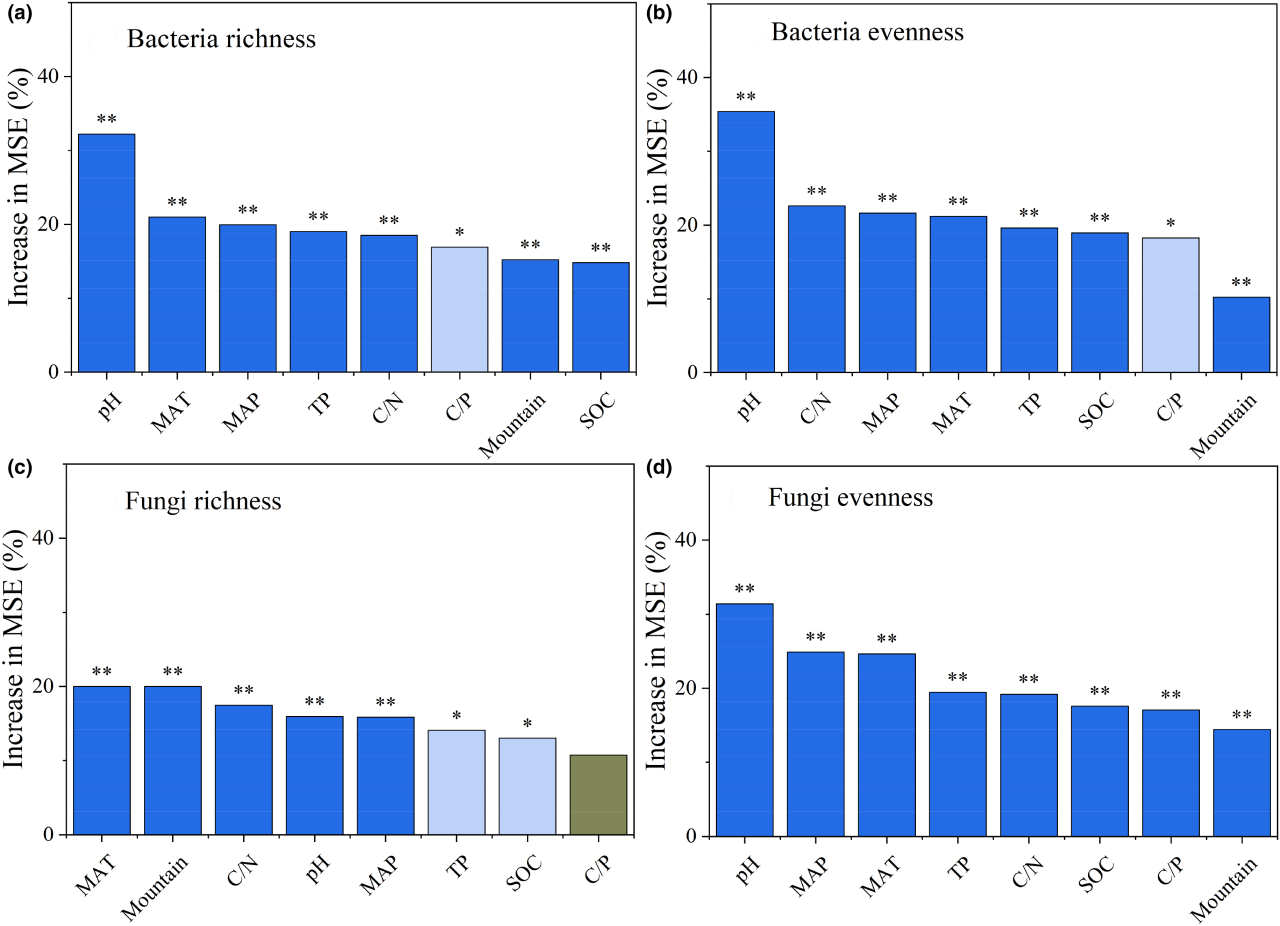
The environmental factors related to the richness and evenness of bacteria and fungi identified with random forest (RF). (a) Bacterial richness. (b) Bacterial evenness. (c) Fungal richness. (d) Fungal evenness. C/N, soil organic carbon/total nitrogen; C/P, soil organic carbon/total phosphorus; MAP, mean annual precipitation; MAT, mean annual temperature; SOC, soil organic carbon; TP, total phosphorus. Mountain: the three climate regions as a categorical variable. Significance levels are as follows: gray column, *p* > .05; light blue column, *.01 < *p* < .05; dark blue column, **p < .01.

NMDS analysis shows that samples clustered together at the same elevations and separated among elevations (Figure 6). The relative abundance of bacteria and fungi at the phylum level also varies with elevation (Figure 3 and Figure S2). Associations of environmental variables with richness and evenness were assessed (the random forest analysis, Figure 7), and soil pH was the most important variable in explaining the variations in bacterial richness (32.24%) and evenness (35.42%). For individual bacterial phyla, pH was also the most important edaphic variable (Figure S4). Bacterial richness varied significantly among mountains and correlated also with other environmental variables, such as MAT (21%) and MAP (19.94%). C/N (22.61%) and MAP (21.62%) were also important in explaining bacterial evenness.

**FIGURE 8:**
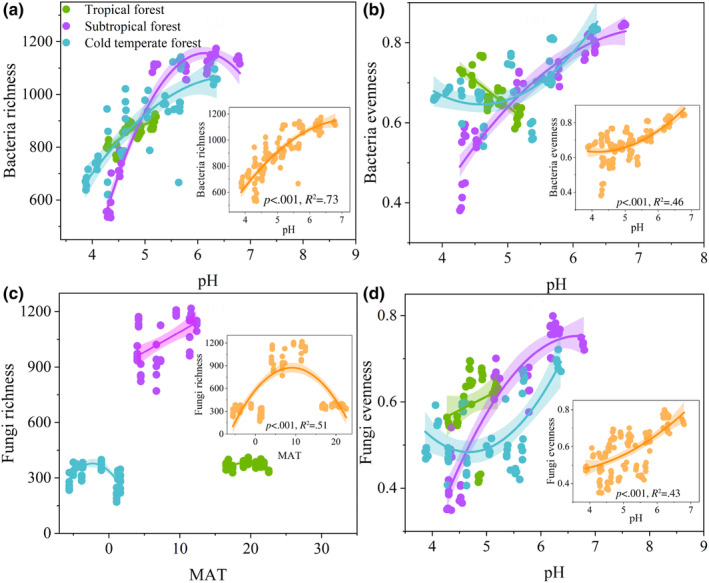
Relationship between bacterial richness and soil pH (a); relationship between bacterial evenness and soil pH (b); relationship between fungal richness and MAT (c); and relationship between fungal evenness and soil pH (d).

For fungi, richness was best explained by MAT (20%), followed by C/N (17.5%) and pH (15.98%). For individual fungal phyla, MAT was the strongest predictors with significant positive effects on individual phyla in subtropical forest and with significant negative effects on individual phyla in cold‐temperate forest (Figure S4). While evenness mostly correlated with pH (31.42%), followed by MAP (24.88%) and MAT (24.63%). Regression analysis showed that the bacterial richness, bacterial evenness, and fungal evenness were significantly correlated with pH. Fungal richness was significantly correlated with MAT (Figure 8).

## 4 DISCUSSION

In our study, we found that elevational patterns of soil microbial diversity vary in three climatic zones and phylum levels. The elevational patterns of microbial richness exhibit inconsistent trends with evenness. The relationship between species richness and evenness showed positive association for the whole bacterial and fungal communities in the subtropical and cold‐temperate forests, but a negative association for bacterial community in the tropical forest. In addition, our analysis demonstrated that soil pH was the most important factor associated with the variations in bacterial richness and evenness, while mean annual temperature explained the major variations in fungal richness. Based on these findings, we emphasize the importance of considering regional climatic conditions and taxonomic resolutions when studying elevation patterns of microbial diversity.

### 4.1 Elevational patterns in soil microbial richness across three climatic zones

Consistent with hypothesis 1, climatic zones affect elevation patterns of bacterial and fungal richness in forest soils. For species richness, the entire soil bacterial community showed inconsistent elevation patterns in climatic zones, with the linear decreasing pattern with elevation in tropical and subtropical forests and asymmetric decreasing pattern in the cold‐temperate forest. Similarly, the elevation patterns in bacterial richness are inconsistent across different climatic regions as we synthesized previous studies (Ji et al., 2022; Nottingham et al., 2018; Wang et al., 2017). Specifically, the linear decrease in bacterial richness with elevation was observed in the tropical and temperature forests, the hump‐shaped pattern showed in some temperate forests, and no obvious change showed in the cold‐temperate forest (Ji et al., 2022; Luo et al., 2019; Nottingham et al., 2018; Ren et al., 2021; Shen et al., 2013). The different elevation patterns in bacterial species richness suggest that different environmental factors (climatic factors, spatial factors, historical factors, and disturbance factors) may be at play in each climatic region (Chalmandrier et al., 2019; Zhu et al., 2020). For bacterial richness, the decreasing trend with elevation supports the water–energy dynamics hypothesis which implies that the nutrient supply along elevation might explain the variation of richness (O'Brien, 2006). Phylum had a significant effect on the elevation pattern of bacterial communities. Among bacterial phyla, approximately 90% of bacterial phyla showed declining species richness with elevation in the subtropical forest, while only 15% and 30% of bacterial phyla showed the declining pattern in the tropical and cold‐temperate forests (Figure S1). Although the declining pattern in the entire bacterial community seems to be typical for species richness, our results suggested that at higher taxonomic resolution levels, more richness–elevation patterns would appear and be dominant, which might explain various richness–elevation relationships in phyla level across different climatic zones.

Similarly, the observed hump‐shaped pattern in fungal richness with elevation in the tropical and cold‐temperate forests and the linear decreasing pattern in the subtropical forest suggest that the elevation pattern of fungal richness might be mainly related to the mid‐domain effect (Colwell et al., 2004). For example, the observed hump‐shaped pattern in ectomycorrhizal fungal richness with elevation in the Mt. Fuji may be due to a combination of environmental filtering and competitive exclusion (Miyamoto et al., 2014), where certain fungal taxa are better adapted to intermediate elevations and competitive interactions limit their abundance at both low and high elevations (Mayor et al., 2017; Nottingham et al., 2015). In contrast, the observed linear decreasing pattern in the subtropical forest may be due to a combination of environmental filtering and facilitation, where certain fungal taxa are better adapted to the specific environmental conditions found at low elevations and positive interactions with other organisms (e.g., mycorrhizal associations) facilitate their establishment and growth (Pellissier et al., 2014; Weiser et al., 2018). In addition, at phyla level, fungal richness–elevation relationships become weaker and more complex and are associated with climatic zones. Overall, these results highlight the importance of considering regional climatic conditions and taxonomic resolutions when studying elevational patterns in biodiversity.

In this study, bacteria (decreased) and fungi (hump‐shaped) showed inconsistent richness elevational pattern in tropical and cold‐temperate forest. There are several possible reasons why bacteria and fungi exhibit different elevational patterns of richness (Bahram et al., 2018; Peay et al., 2017; Shen et al., 2020). One possible explanation is that bacteria and fungi have distinct physiological characteristics that can lead to variations in their responses to elevation‐related environmental factors, such as temperature, substrate availability, and elevation‐associated stressors (Louca et al., 2018; Peay et al., 2017). For instance, fungi tend to be more resistant to changes in temperature and substrate availability, due to their larger size and dependence on external soil nutrients (Männistö et al., 2018). The peak of fungal richness in mid‐elevation in tropical and cold‐temperate forests may result from a combination of temperature and substrate availability that maintain relatively suitable conditions at mid‐elevation (Ren et al., 2021). Another possible explanation is that variations in microbial interactions and community assembly along elevation gradients could lead to inconsistent shifts in richness patterns for bacteria and fungi (Ji et al., 2022). For instance, some studies have suggested that bacteria have higher dispersal rates and lower endurance than fungi, which could make the change of richness and environmental gradient more consistent (Ji et al., 2022; Walters et al., 2022). In contrast, fungi may have lower dispersal rates and high tolerance, which could make fungi persist in high resource‐rich but volatile environments, such as at middle or high elevation (Margesin et al., 2009). In addition, microhabitat specificity may determine fungal distribution patterns (Miyamoto et al., 2014). Some fungal species might be adapted to specific microenvironments, such as mycorrhizal fungi depends on the host plant within certain elevation ranges (Truong et al., 2019).

In summary, the inconsistency in microbial diversity of climatic zones’ responses to elevation gradient implies microbial community responses to future climate changes might be localized‐dependent nature (Wang et al., 2017). It highlights the importance of localized approaches in managing and mitigating the effects of climate change on ecosystems (Choi et al., 2021). In addition, the inconsistency between richness–elevation patterns at the whole community level and the phylum level emphasizes the need to consider multiple taxonomic scales when studying microbial diversity and its responses to environmental gradients. It also highlights the intricate relationships among microbial functional groups and their roles in maintaining ecosystem functionality along elevation gradients (Escalas et al., 2019).

### 4.2 Elevational patterns in soil microbial evenness across three climatic zones

Consistent with hypothesis 1, climatic zones affect the elevation patterns of bacterial and fungal evenness in forest soils. The evenness of the bacterial community increased linearly with elevation in the tropical forest but exhibited a hump‐shaped pattern in the subtropical and cold‐temperate forests. Similarly, the evenness of the fungal community showed a hump‐shaped pattern with elevation increase in all three climatic regions. The diversified evenness–elevation patterns in soil bacterial community were consistent with that of aquatic bacteria across different climatic regions (Nogues‐Bravo et al., 2008). One possible explanation for the contrast elevational patterns in climatic zones is the effects of temperate on the distribution and abundance of soil bacterial communities (Zhang et al., 2022). For instance, in the tropical forest, the high temperature and precipitation levels may favor certain bacterial taxa that are adapted to such conditions, resulting in a more even distribution of species across the elevation gradient. In contrast, the colder and drier conditions in the subtropical and cold‐temperate forests may select for different bacterial taxa, resulting in a less even distribution of species along the elevation gradient (Ma et al., 2016). In addition, as an important boundary, the tree line in tropical forest is higher than that in subtropical and cold‐temperate forests, which makes the difference in forest microbial composition relatively small in tropical region, and the evenness of microorganisms increases with the elevation (Shen et al., 2019). Finally, it is also possible that the observed differences in the evenness–elevation of bacteria and fungi may be due to scale effects (Chen et al., 2015; Nogues‐Bravo et al., 2008). For example, some microbial taxa may be close to certain vegetation and habitat or may be more sensitive to variations in temperature or moisture (Li et al., 2016). The range of environmental factors in the elevation gradient can affect the extent and magnitude of microbial community change (Wang et al., 2017).

### 4.3 Relationship between soil microbial richness and evenness

The relationship between richness and evenness is an important ecological concept, as it helps to understand the community structure and diversity of an ecosystem (Wilsey et al., 2005). Consistent with hypothesis 2, there is no consistent pattern of RRE across climatic zones, trophic levels, and taxonomic levels. In our study, we found different relationships between richness and evenness in bacteria and fungi, and also across different climatic zones and taxonomic levels. In the tropical forest, we observed a negative relationship between richness and evenness in bacteria, indicating that some bacterial species were more dominant than others. In contrast, fungi in the tropical forest showed a positive quadratic relationship between richness and evenness, suggesting that the fungal community was more evenly distributed across different species. In the subtropical and cold‐temperate forests, we observed a positive relationship between richness and evenness in both bacteria and fungi. This suggests that the community structure in these forests is more balanced, with similar levels of abundance for different species.

At the phylum level, we found that 83% of phyla showed a positive RRE. This is consistent with the results in stream biofilm microorganisms (Wang et al., 2017), but in contrast to the prediction of ‘species–energy theory’ (Evans et al., 2005). The negative RRE of bacteria occurs only in tropical forest. The differences in research results may come from the research background and the differences between different taxonomies. Fungi showed more positive RREs than bacteria. This may be due to the stronger adaptability in fungi (Truong et al., 2019). Further, bacterial relative abundance did not affect RRE, but higher relative abundance of fungi was linked to higher RRE. This may be explained by the high adaptability of fungi, which enables them to colonize and thrive in different environments (Wang et al., 2021). Fungi with higher relative abundance are also less prone to extinction due to their larger populations and increased production of propagules (Finlay, 2002). These results suggest that fungal communities have greater stability (Wang et al., 2021).

### 4.4 Explaining the elevational patterns in soil microbial richness and evenness

Inconsistent with hypothesis 3, the results showed that soil factor (pH) controls the elevation pattern of soil bacterial richness, bacterial evenness, and fungal evenness, while climatic factor (MAT) controls the elevation pattern of fungal richness. In mountain ecosystems, soil pH is the primary environmental factor driving bacterial richness and evenness (Shen et al., 2019). Bacterial richness and evenness increase with increasing pH and reach a maximum at neutral pH (Figure 8), which is consistent with the findings of previous studies (Fierer & Jackson, 2006; Shen et al., 2019). Deviation from the neutral pH value may exert physiological constraints and the energy costs on microorganisms, which may limit the possibility of multiple species living in the same niche (Luo et al., 2019; Tripathi et al., 2012). Bacterial richness and evenness are mainly controlled by soil pH, which is consistent with the results of other microbial diversity studies (Luo et al., 2019; Shen et al., 2019; Wang et al., 2017), but the control factors for fungal richness and evenness are different. MAT is the most significant variable affecting fungal richness, which supports the ‘temperature hypothesis’ (Ren et al., 2021). Studies have shown that climatic factors, particularly temperature, drive changes in microbial diversity across large geographical ranges by altering microbial metabolism and growth (Liu et al., 2022; Zhou et al., 2016). Two mechanisms contributing to the different relative effects of MAT and pH on fungal richness and evenness. First, the weak effect of soil pH on fungal richness may be related to the relative high resource utilization and eco‐physiological adaptation in fungi (Barnard et al., 2013). For example, the optimal pH range of fungal communities was more comprehensive (pH 5–9) than that of bacteria (pH 4–7; Nevarez et al., 2009); thus, fungal richness may be less sensitive to pH than fungal evenness. Second, fungal richness was also related to soil organic carbon and C/N ratio, and the accumulation and decomposition of soil organic carbon in the soil were determined by MAT (Tan et al., 2021). This may have resulted in the control of MAT on fungal richness. In sum, the relative importance of pH and MAT in driving microbial richness differs for bacteria and fungi. Considering that MAT is susceptible to climate change influences, temperature fluctuations associated with global warming may lead to changes in patterns of fungal community richness. Conversely, bacteria may exhibit diverse responses (Peay et al., 2017). Importantly, existing carbon cycle models often neglect the diverse nature of microbial reactions (Treseder et al., 2012). Researchers studying soil microbial responses to changing environmental conditions should consider both pH and MAT as key variables. This could guide more accurate predictions in climatic model (Treseder et al., 2012).

## 5 CONCLUSION

The study found that there was no consistent pattern of species richness and evenness of soil bacteria and fungi across the three climatic zones studied. The richness of the entire bacterial community showed a significant decreasing pattern across the climatic zones, while the evenness of the bacterial community did not show a consistent pattern. The entire fungal community in the subtropical forest showed decreasing pattern in richness and evenness, while hump‐shaped pattern was observed in the tropical and cold‐temperate forests. The bacterial and fungal community in the subtropical and cold‐temperate forests showed a positive RRE across climatic zones, while decreasing RRE was observed for bacterial community in the tropical forest. Soil pH explained most of the variation in bacterial richness and evenness, as well as fungal evenness, while mean annual temperature (MAT) explained most of the variation in fungal richness. The climatic zones explained the largest variations of RRE. These findings suggest that predicting the effects of future climatic changes on soil microbial communities requires a more comprehensive understanding of microbial responses across geological spatial and taxonomic levels.

## AUTHOR CONTRIBUTIONS

**Zhengyi Huang:** Conceptualization (equal); data curation (equal); formal analysis (equal); methodology (equal); resources (equal); software (equal); supervision (equal); visualization (equal); writing – original draft (equal); writing – review and editing (equal). **Yangui Su:** Conceptualization (equal); formal analysis (equal); funding acquisition (equal); supervision (equal); visualization (equal); writing – original draft (equal); writing – review and editing (equal). **Sinuo Lin:** Conceptualization (equal); data curation (equal); methodology (equal); software (equal); validation (equal); visualization (equal). **Guopeng Wu:** Conceptualization (equal); formal analysis (equal); supervision (equal); validation (equal); visualization (equal). **Hao Cheng:** Conceptualization (equal); formal analysis (equal); validation (equal); visualization (equal). **Gang Huang:** Conceptualization (equal); data curation (equal); formal analysis (equal); funding acquisition (equal); project administration (equal); software (equal); writing – original draft (equal); writing – review and editing (equal).

## FUNDING INFORMATION

This work was supported by the National Natural Science Foundation of China (Nos. 32171643, 41671115, U1703332).

## CONFLICT OF INTEREST STATEMENT

The authors declare no conflict of interests.

## Supporting information


Appendix S1.

## Data Availability

The data that support the findings of this study are available from the Dryad Digital Repository https://doi.org/10.5061/dryad.7h44j100w. The 16S and ITS sequences were submitted to the National Genomics Data Center Nucleotide Sequence Database (Accession Number: PRJCA015582).
