# Supplementary material for: Elevational patterns of microbial species richness and evenness across climatic zones and taxonomic scales
Source: Ecol Evol. 2023 Oct 9;13(10):e10594. doi: 10.1002/ece3.10594 (PMC10560872; doi:10.1002/ece3.10594)
Supplement: Supplementary file 1 — Appendix S1. [file ECE3-13-e10594-s001.docx]

**SUPPORTING INFORMATION**

**Table S1** The geographic location, temperature, precipitation, soil type and dominant vegetation of the sampling sites.

| **Sampling sites** | **Latitude and longitude** | **Elevation (m)** | **MAT (℃)** | **MAP(mm)** | **Soil type** | **Vegetation** | **Main plant species** |
| --- | --- | --- | --- | --- | --- | --- | --- |
| JFL-1 | 18°42.1′N, 108°50.2′E | 409 | 22.55 | 1329 | Latosol | Tropical evergreen seasonal rainforest | *Terminalia hainanensis、Lannea grandis、Taxotrophis aquifolioides* |
| JFL-2 | 18°42.0′N, 108°50.5′E | 524 | 21.86 | 1349 | Latosol | Tropical evergreen seasonal rainforest | *Terminalia hainanensis、Lannea grandis、Taxotrophis aquifolioides* |
| JFL-3 | 18°42.0′N, 108°51.1′E | 595 | 21.44 | 1422 | Latosol | Tropical evergreen seasonal rainforest | *Terminalia hainanensis、Lannea grandis、Taxotrophis aquifolioides* |
| JFL-4 | 18°42.2′N, 108°52.1′E | 815 | 20.73 | 1501 | yellow soil | Tropical mountain rain forest | *Terminalia hainanensis、Lannea grandis、Taxotrophis aquifolioides* |
| JFL-5 | 18°42.3′N, 108°52.3′E | 891 | 19.66 | 1501 | yellow soil | Tropical mountain rain forest | *Mallotus hookerianus、Cyclobalanopsis patelliformis* |
| JFL-6 | 18°42.7′N, 108°52.6′E | 1018 | 18.89 | 1574 | yellow soil | Tropical mountain rain forest | *Syzygium jambos* |
| JFL-7 | 18°43.0′N, 108°52.5′E | 1259 | 17.45 | 1544 | yellow soil | Mountainous semi deciduous rainforest | *Mallotus hookerianus、Cyclobalanopsis patelliformis* |
| JFL-8 | 18°43.0′N, 108°52.5′E | 1357 | 16.86 | 1574 | yellow soil | Mountainous semi deciduous rainforest | *Cyclobalanopsis jenseniana、Cinnamomum parthenoxylon* |
| JFL-9 | 18°43.0′N, 108°52.2′E | 1410 | 16.54 | 1544 | yellow soil | Mountain coppice | *Gordonia axillaris、Rhododendron klossii Ridl* |
| SNJ-1 | 31°41.0′N, 110°11.2′E | 864 | 12.4 | 976 | Udalfs | Evergreen broad-leaved forest | *Spruce、Liquidambar formosana、Oak、oil-tea camellia、Cyclobalanopsis glauca、Palm* |
| SNJ-2 | 31°41.4′N, 110°11.6′E | 1282 | 11.6 | 1274 | Udalfs | Mixed-deciduous and evergreen broad-leaved forest | *Chestnut tree、Oak、Ramie、Mint、Maple、Liquidambar formosana* |
| SNJ-3 | 31°41.4′N, 110°11.6′E | 1300 | 11.4 | 1123 | Udalfs | Mixed-deciduous and evergreen broad-leaved forest | *Chestnut、Birch* |
| SNJ-4 | 31°41.7′N, 110°11.9′E | 1518 | 9.5 | 1006 | Udalfs | Mixed-deciduous and evergreen broad-leaved forest | *Phyllostachys pubescens 、Pinus armandii、Fern* |
| SNJ-5 | 31°41.8′N, 110°12.0′E | 1765 | 7.3 | 1519 | Hapludalfs | Mixed-coniferous and deciduous forest | *Pinus armandii、Oak* |
| SNJ-6 | 31°41.9′N, 110°12.1′E | 1892 | 6.7 | 1590 | Hapludalfs | Mixed-coniferous and deciduous forest | *Pinus armandii、Quercus、Star anise maple、Pentagonal maple、Smilax China、Hamamelis、Dioscorea nipponica、Runnan、Porcupine thorn* |
| SNJ-7 | 31°42.2′N, 110°12.3′E | 2400 | 4.2 | 1891 | Hapludalfs | Coniferous forest | *Fir forest、Schisandra chinensis、Small red hemp、Kudzu bat、Blood water herb、Ramie、Tarragon、Smilax China、Porcupine thorn、Runnan、Dioscorea nipponica、Hamamelis* |
| SNJ-8 | 31°42.3′N, 110°12.4′E | 2699 | 4.5 | 2062 | Hapludalfs | Subalpine dwarf-shrub | *Azalea、Bamboo* |
| SNJ-9 | 31°42.3′N, 110°12.4′E | 2856 | 4.0 | 2500 | Hapludalfs | Subalpine dwarf-shrub | *Rhododendron、Spruce、Tarragon* |
| XAL-1 | 47°10.0′N, 128°51.0′E | 331 | 1.10 | 627 | brown soil | Deciduous mixed forest | *Birch、Spruce、Ebony、Poplar* |
| XAL-2 | 47°12.0′N, 128°53.0′E | 376 | 1.62 | 628 | brown soil | Coniferous forest | *Fir、Spruce、Abies odorifera、Acer pentagonum、Manchurian ash、Scallion wood* |
| XAL-3 | 47°10.0N, 128°53.0′E | 398 | 1.62 | 619 | brown soil | Coniferous forest | *Korean pine、Mongolian oak、Manchurian as* |
| XAL-4 | 47°11.1N, 128°53.1′E | 408 | 1.27 | 626 | brown soil | Coniferous forest | *Larix gmelinii、Beech、Korean pine、* |
| XAL-5 | 50°25.0′N, 124°07.0′E | 425 | -1.06 | 510 | brown soil | Coniferous and broad-leaved mixed forest | *Aspen、Birch、Larch、Quercus mongolica、Ulmus pumila* |
| XAL-6 | 50°25.0′, 124°07.1′E | 429 | -1.06 | 510 | brown soil | Broad-leaved forest | *Mongolian oak* |
| XAL-7 | 51°46.0′N, 123°34.1′E | 612 | -3.70 | 517 | brown soil | Coniferous and broad-leaved mixed forest | *Larch、Birch* |
| XAL-8 | 51°42.0′N, 123°35.1′E | 671 | -3.83 | 541 | brown coniferous forest soil | Coniferous forest | *Larix gmelinii* |
| XAL-9 | 51°38.0′N, 123°32.1′E | 890 | -4.65 | 541 | brown coniferous forest soil | Coniferous and broad-leaved mixed forest | *Larch、Birch、Aspen* |
| XAL-10 | 51°37.0′N, 123°32.0′E | 1186 | -5.01 | 556 | brown coniferous forest soil | Coniferous forest | *Pinus sylvestris var. mongolica* |
| XAL11 | 51°37.0′N, 123°32.1′E | 1261 | -5.58 | 572 | brown coniferous forest soil | Coniferous forest | *Pinus pumila elfin* |


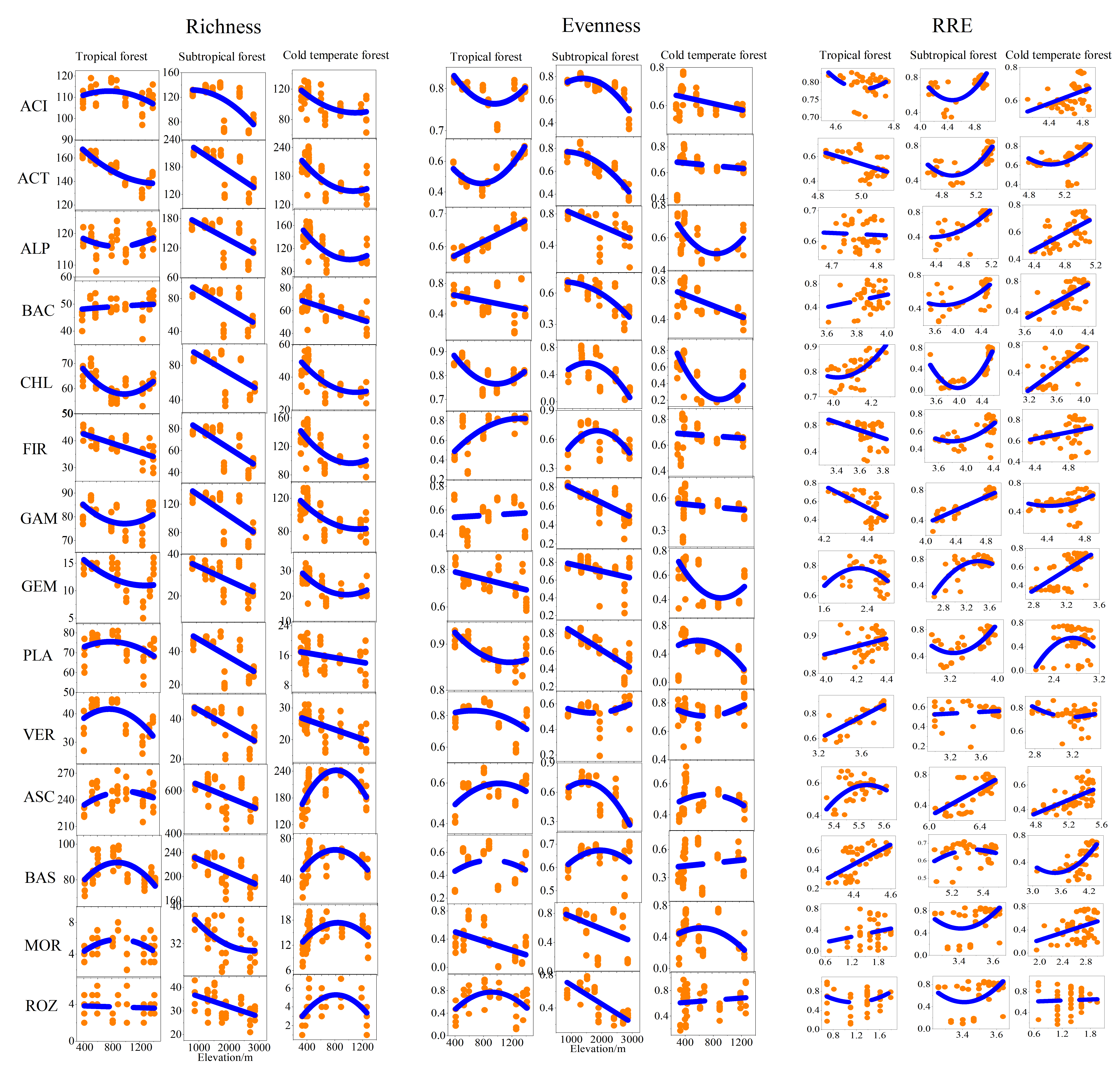


**Fig. S1.** Elevational patterns in biodiversity of microbial phyla. The trends along elevations were modeled with both linear and quadratic models. The better model was selected based on the lower value of Akaike’s information criterion, and is shown as solid. Non-significant trends for both models are shown with dotted lines. ACI, Acidobacteria; ACT, Actinobacteria; ALP, Alphaproteobacteria; BAC, Bacteroidetes; CHL, Chloroflexi; FIR, Firmicutes; GAM, Gammaproteobacteria; GEM, Gemmatimonadetes; PLA, Planctomycetes; VER, Verrucomicrobia; ASC, Ascomycota; BAS, Basidiomycota; MOR, Mortierellomycota; ROZ, Rozellomycota.


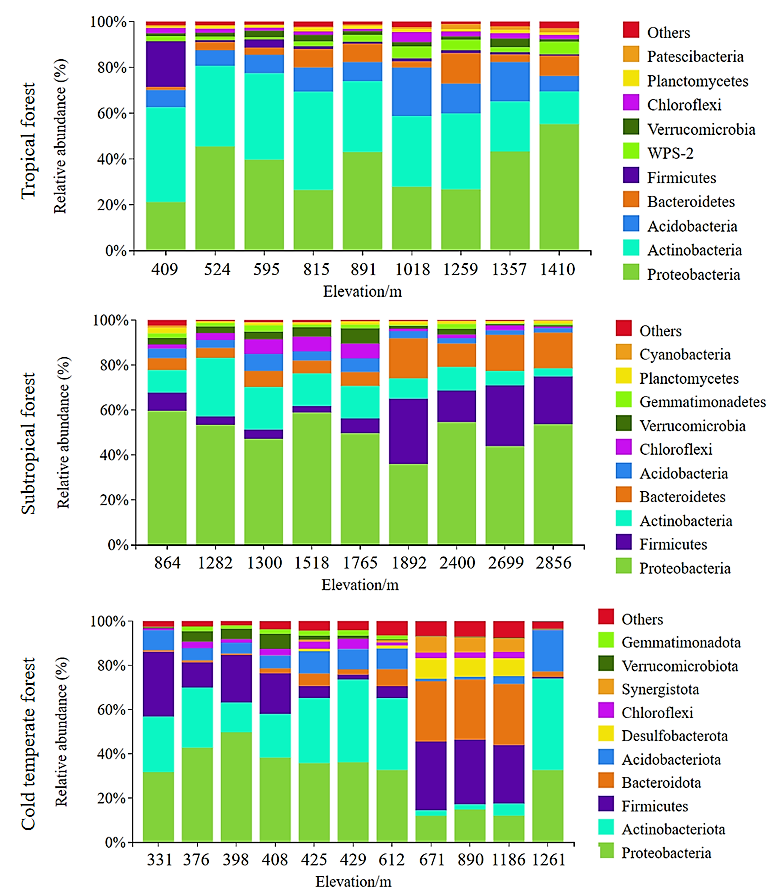


**Fig. S2.** Change of bacterial communities relative abundance along the elevation in three climatic zone.


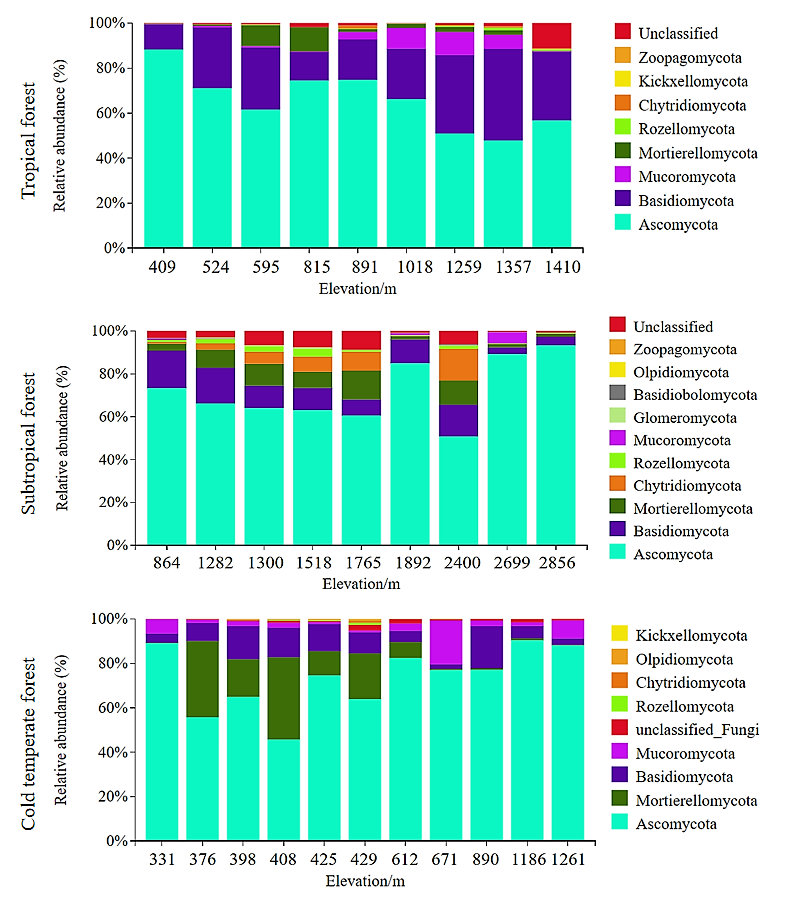


**Fig. S3.** Change of fungi communities relative abundance along the elevation in three climatic zone.


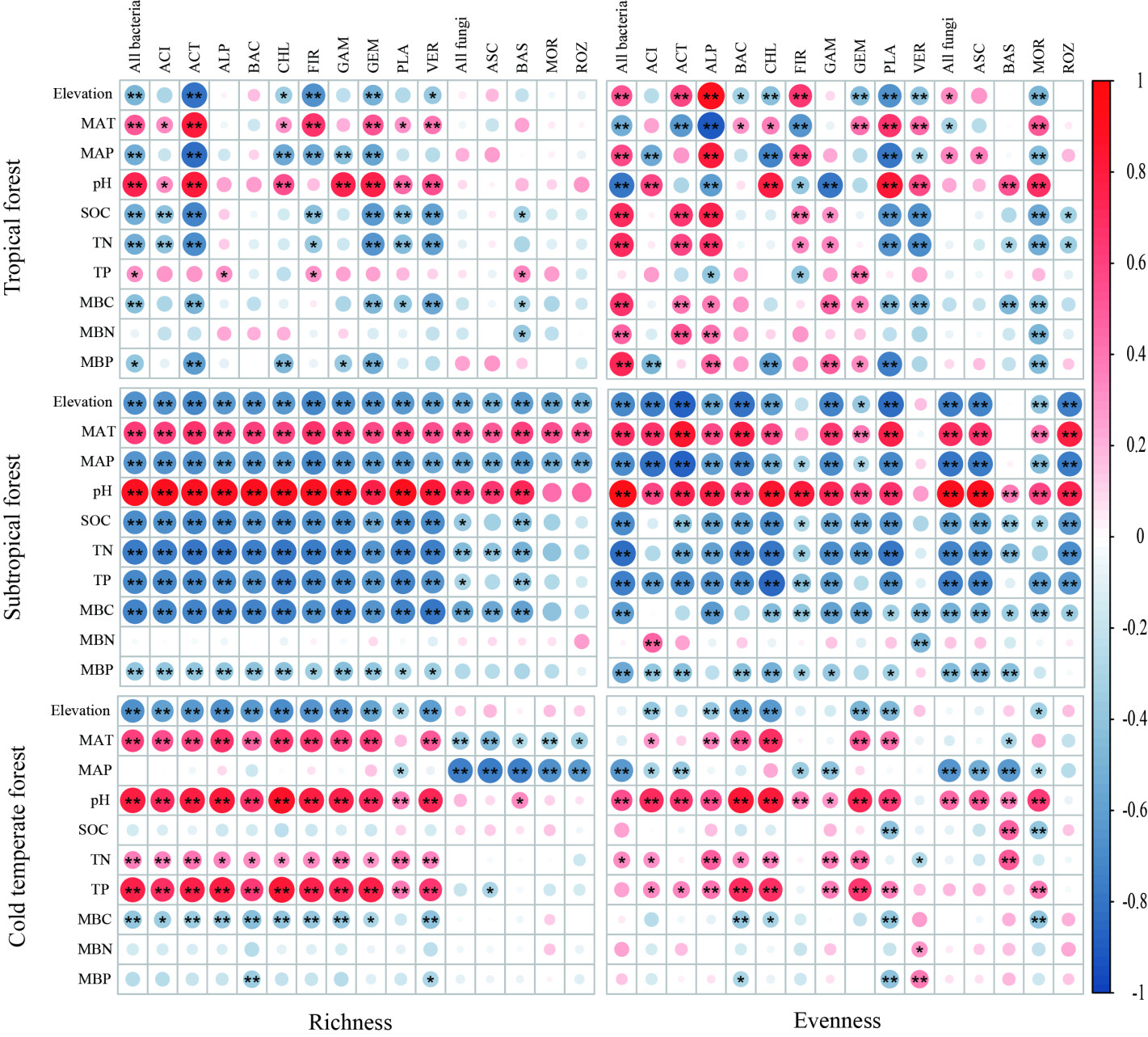


**Fig. S4.** Pearson correlation between biodiversity and environmental variables in three climatic zone. MAT, mean annual temperature; MAP, mean annual precipitation; SOC, soil organic carbon; TN, total nitrogen; TP, total phosphorus; MBC, microbial biomass carbon; MBN, microbial biomass nitrogen; MBP, microbial biomass phosphorus. ACI, Acidobacteria; ACT, Actinobacteria; ALP, Alphaproteobacteria; BAC, Bacteroidetes; CHL, Chloroflexi; FIR, Firmicutes; GAM, Gammaproteobacteria; GEM, Gemmatimonadetes; PLA, Planctomycetes; VER, Verrucomicrobia; ASC, Ascomycota; BAS, Basidiomycota; MOR, Mortierellomycota; ROZ, Rozellomycota.
